# Comparative analysis of semaglutide induced adverse reactions: Insights from FAERS database and social media reviews with a focus on oral vs subcutaneous administration

**DOI:** 10.3389/fphar.2024.1471615

**Published:** 2024-10-22

**Authors:** Jing Zhang, Xiaofen Wang, Yiting Zhou

**Affiliations:** ^1^ Department of Pharmacy, Sir Run Run Shaw Hospital, School of Medicine, Zhejiang University, Hangzhou, Zhejiang, China; ^2^ Clinical Laboratory, Sir Run Run Shaw Hospital, School of Medicine, Zhejiang University, Hangzhou, Zhejiang, China

**Keywords:** pharmacovigilance, semaglutide, disproportionality analysis, FAERS, social media reviews

## Abstract

**Background:**

Compared to alternative weight-loss strategies and medications, semaglutide stands out for its convenience and efficacy, resulting in a significant increase in prescriptions and raising public safety concerns. Furthermore, the safety profiles of its oral and subcutaneous formulations require further examination.

**Objective:**

Our goal is to investigate the potential safety risks associated with semaglutide by analyzing data from the FAERS database and social media. Additionally, we aim to compare the adverse drug reaction (ADR) signals between the oral and subcutaneous administration routes of semaglutide.

**Methods:**

We collected semaglutide-related reports from the FAERS database spanning Q1 2018 to Q2 2023, and patient reviews on WebMD and AskaPatient up to 20 July 2023. Following data extraction and cleansing, we conducted descriptive analyses of demographic characteristics. Subsequently, we calculated adverse drug reaction (ADR) signals using the reporting odds ratio (ROR).

**Results:**

We identified 19,289 and 422 semaglutide-related adverse drug events (ADEs) reported in the FAERS database and online patient reviews, respectively. Gastrointestinal disorders emerged as the most commonly reported System Organ Class (SOC) in both datasets. Predominant Preferred Terms (PTs) included nausea, vomiting, and diarrhea. Serious outcomes constituted 3.07% and 2.25% of all cases for oral and subcutaneous semaglutide, respectively. At the SOC level, gastrointestinal disorders accounted for 30.19% of total ADEs in oral semaglutide, slightly surpassing the 27.76% in subcutaneous semaglutide. The median onset for gastrointestinal PTs was 4 days in both oral (Q1: 1, Q3: 32) and subcutaneous (Q1: 1, Q3: 35) formulations. Noteworthy, new serious adverse event (AE) signals were identified, including hemorrhagic diarrhea (ROR: 3.69), hepatic pain (ROR: 4.20), abnormal hormone levels (ROR: 6.51), and pancreatic failure (ROR: 36.34) in subcutaneous semaglutide, and Dupuytren’s contracture (ROR: 46.85) in oral semaglutide.

**Conclusion:**

Our study delineates the safety profile of semaglutide using data from the FAERS database and social media. And identified novel ADR signals specific to oral and subcutaneous forms of semaglutide.

## 1 Introduction

Semaglutide, which belongs to the family of incretin glucagon-like peptide-1 receptor agonists (GLP-1 RAs), is a peptide approved for the long-term treatment of type 2 diabetes (T2DM) and obesity. As GLP-1 agonists, semaglutide can mimic the effects of GLP-1, such as increasing insulin production, thus decreasing blood glucose levels and slowing gastric emptying, serving as a weight-loss medication. Semaglutide significantly decreased HbA1c levels by 1.55% at a dosage of 1.0 mg (Sorli et al., 2017) and improved cardiovascular outcomes in patients with type 2 diabetes (Marso et al., 2016). Semaglutide has demonstrated the highest percentage of weight loss in obese adults without type 2 diabetes, with reductions of approximately 15% of initial weight at 68 weeks (Wilding et al., 2021). The most common adverse events (ADE) are gastrointestinal issues, and the risks of pancreatitis, kidney failure, and medullary thyroid carcinoma cannot be ignored (Smits and Van Raalte, 2021).

Generally, after marketing approval, pharmacovigilance analysis could be conducted by utilizing the ADE data from public database such as the FAERS database. Several studies have reported associations between semaglutide and ADR signals related to gastrointestinal, retinal, and tumor adverse events (Shu et al., 2022; Fadini et al., 2018; Yang et al., 2022). In various pharmacovigilance studies, semaglutide’s safety profile has been evaluated against other GLP-1 RAs. Semaglutide-associated overall tumor (Yang et al., 2022) and the risk of suicidality or self-injury was similar to the overall GLP-1RA (Chen et al., 2023). While semaglutide exhibited the highest risk of nausea, diarrhea, vomiting, and constipation (Liu et al., 2022) and tended to have a higher susceptibility to metabolic and nutritional AEs (He et al., 2024). Additionally, a separate analysis assessed the risk of pancreatitis and found that semaglutide had a high ROR, similar to liraglutide, indicating a higher risk compared to exenatide, which served as the reference drug (Alenzi et al., 2024).

Online patient reviews provide earlier insights into certain ADEs or additional perspectives on ADEs from the patient’s viewpoint, which can be utilized for pharmacovigilance purposes (Pierce et al., 2017; Song et al., 2021). Furthermore, as semaglutide’s popularity increases for weight loss, the heightened demand has resulted in supply issues across various licensed products, including those for diabetes treatment. The widespread use of semaglutide has sparked frequent heated discussions, particularly regarding its safety, on public health forums and social media. However, a comprehensive safety profile of semaglutide, including its effects on all body systems, integrating data from the FAERS database and social networks, is still lacking.

Semaglutide is available in both injectable and oral formulations. The dose for treating T2DM with once-weekly subcutaneous semaglutide is 1.0 mg, while the weight management dose is 2.4 mg injected subcutaneously once weekly. The oral version has a maximum daily dose of 14 mg, approved for treating T2DM. Diabetic patients have the option of selecting either subcutaneous injections of 1.0 mg or oral medications, both of which may potentially contribute to weight loss effects in patients. Although there are no head-to-head studies comparing the approved doses of oral semaglutide (7 mg and 14 mg) with once-weekly subcutaneous semaglutide (0.5 mg and 1.0 mg). In a systematic review and network meta-analysis, reductions in HbA1c with once-weekly semaglutide 1 mg were numerically greater than with orally administered semaglutide 14 mg, while the efficacy in weight loss was nearly identical. Regarding the safety outcomes of gastrointestinal adverse drug events (ADEs), orally administered semaglutide showed statistically similar associations with the injectable formulation (Nuhoho et al., 2019). However, detailed safety profiles for different formulations have not been fully investigated.

The present study aimed to identify new signals indicating potential safety risks associated with semaglutide in the FAERS database and on social media platforms. Additionally, it aimed to compare the differences in adverse drug reaction (ADR) signals between oral and subcutaneous administration of semaglutide.

## 2 Materials and methods

### 2.1 Data source

The data sources for our study included the US Food and Drug Administration Adverse Event Reporting System (FAERS) and social media reviews. The FAERS database is one of the largest openly accessible databases, receiving spontaneous reports from global pharmaceutical manufacturers, consumers, pharmacists, doctors, patients, etc (Zhou et al., 2023). It contains seven different tables covering information such as demographics, drugs, reactions, outcomes, therapy, report sources, and indications. We collected information on ADEs associated with semaglutide from the FAERS database for the period spanning from 2018Q1 to 2023Q2. Nowadays, drug safety information is available on websites such as WebMD, AskaPatient, and Drugs.com in the form of patient reviews. In a few words, the patient shares his/her personal experience with efficacy, duration, adverse events, patient satisfaction evaluations and other issues (Lardon et al., 2015). In our study social media reviews contained two websites, WebMD (https://www.webmd.com) and Ask a Patient (https://www.askapatient.com). Examples of patients reviews were shown in Supplementary Table S1. Only reviews describing ADRs were collected for further analysis. For text mining, data of patient reviews were downloaded using the Python-based library Beautiful Soup (Song et al., 2021). The research of semaglutide associated ADRs in those websites were conducted on 20th,July 2023.

### 2.2 Data extraction and cleaning

ADE reports were identified, and duplicated data were eliminated based on primary ID and reported date in the FAERS database. The report with the largest primary ID and the latest reported date was selected for further analysis. (Zhang et al., 2022). All cases with “semaglutide” as the primary suspected drug were included in our study. ADRs are classified and described according to the preferred term (PT) and system organ class (SOC). PT is variable for standard AEs and SOC belongs to the top level in the International Medical Dictionary for Regulatory Activities (MedDRA) version 25.0 (Hu et al., 2021). Online patient reviews of semaglutide of both oral and subcutaneous forms were collected from the WebMD and AskaPatient website. Reviews that were not relevant to drug-related adverse events, including drug prescription advertisements or unusual drug exposure duration were eliminated. Finally, each drug review was manually transformed from descriptions to PT based on MedDRA terminology by Jing Zhang and Yiting Zhou independently. In case of any inconsistencies, Jing Zhang and Yiting Zhou would make a decision after discussion, referring to previous reports.

### 2.3 Comparative analysis

Semaglutide is currently available in two formulations: tablet (Rybelsus) and injection (Wegovy and Ozempic). These two different dosage forms were identified based on drugname, route, dose_form or dose_freq. If all of the information regarding trade name, route of administration, dose form, or dosing frequency were missing, the case would not be included in the comparative analysis. A Venn diagram was used to obtain the difference of PTs between two formulations by using the ‘VennDiagram’ package of R program (Jia et al., 2021).

### 2.4 ADR signal detection

We performed a disproportion analysis of the unique PTs reported between various formulations in FAERS database to identify the difference in ADR signal. Disproportion analysis were based on the 2 × 2 four-grid table (Supplementary Table S2), wherein the ratio of suspected AEs associated with the target drug to AEs caused by other drugs in the database was calculated. If the calculated value exceeds the threshold, a statistical association between the drug and the suspected AE is considered. The reporting odds ratio (ROR) method can estimate the relative risk, which is determined by the lower limit of the 95% confidence interval (CI). When the lower limit of the 95% CI is greater than 1.0, a ADR signal was detected. The ROR was calculated using the following formula: ROR = (a/c)∕(b/d), and the 95% CI was determined using the formula: 95% CI = e^ln(ROR)±1.96(1/a+1/b+1/c+1/d)^0.5^. The analysis was performed with R version 4.2.2. A forest plot was generated to illustrate the detection of adverse drug reaction (ADR) signals using the ‘forestplot’ package in the R programming language after uploading the calculation results of ROR.

## 3 Results

### 3.1 Descriptive results

There were 19,289 semaglutide-related ADEs reported in the FAERS database and 422 in online patient reviews. As shown in Table 1, the number of reports generally increased over time. The median age of patients reported in the FAERS database was 62. The age group of 35–65 reported the highest number of adverse reactions, accounting for 29.62% of the total reports. In online patient reviews, the age group of 35–65 reported 312 cases, representing 73.93% of the total reports. The majority of cases were female, comprising 60.6% and 75.36% of the total cases, respectively. The majority of reported cases originated from the United States, followed by Great Britain and Canada.

**TABLE 1:** Characteristics of patients from FAERS^a^ and social media.

Characteristics	FAERS n (%)	Social media n (%)
Total	19,289	422
Year		
2018	1,114 (5.77%)	2 (0.47%)
2019	1,357 (7.03%)	20 (4.74%)
2020	2,779 (14.41%)	99 (23.46%)
2021	4,075 (21.13%)	126 (29.86%)
2022	6,207 (32.18%)	106 (25.12%)
2023 season 1 and 2	3,757 (19.48%)	69 (16.35%)
Age	62 (median)	
less than 35 years old	484 (2.51%)	25 (5.92%)
35–65 years old	5,713 (29.62%)	312 (73.93%)
more than 65 years old	4,106 (21.29%)	80 (18.96%)
Unknown	8,986 (46.59%)	5 (1.18%)
Sex		
male	6,896 (35.75%)	93 (22.04%)
female	11,689 (60.6%)	318 (75.36%)
Unknown	704 (3.65%)	11 (2.6%)
Country		
UNITED STATES	16,589 (86%)	N/A^b^
GREAT BRITAIN	372 (1.93%)	N/A
CANADA	370 (1.92%)	N/A
JAPAN	345 (1.79%)	N/A
FRANCE	176 (0.91%)	N/A
BRAZIL	159 (0.82%)	N/A
DENMARK	157 (0.81%)	N/A
AUSTRALIA	150 (0.78%)	N/A
ISRAEL	86 (0.45%)	N/A
SWEDEN	80 (0.41%)	N/A

^a^ FAFAERS: US, Food and Drug Administration (FDA) adverse event reporting system.

^b^ N/A: not applicable.

### 3.2 Comparison of AEs reported in FAERSa and social media

Subsequently, we analyzed the SOC in the FAERS database and online patient reviews. The SOC results in Table 2 revealed greater diversity in the FAERS database, with 27 SOCs reported compared to 18 SOCs in social media. Gastrointestinal disorders were the most frequently reported SOC in both datasets, followed by general disorders and administration site conditions. Most SOC data were similar between the two datasets, except for respiratory, thoracic, and mediastinal disorders, which were reported more frequently in social media, and investigations, which were reported more frequently in the FAERS database.

**TABLE 2:** System organ class reported in FAERS^a^ and social media.

FAERS		Social media	
SOC^b^	n (%)	SOC	n (%)
Gastrointestinal disorders	15,704 (28.02%)	Gastrointestinal disorders	864 (63.62%)
General disorders and administration site conditions	6,787 (12.11%)	General disorders and administration site conditions	128 (9.43%)
Injury, poisoning and procedural complications	6,093 (10.87%)	Respiratory, thoracic and mediastinal disorders	114 (8.39%)
Investigations	4,780 (8.53%)	Nervous system disorders	99 (7.29%)
Nervous system disorders	4,047 (7.22%)	Metabolism and nutrition disorders	72 (5.30%)
Metabolism and nutrition disorders	3,525 (6.29%)	Psychiatric disorders	30 (2.21%)
Skin and subcutaneous tissue disorders	1878 (3.35%)	Musculoskeletal and connective tissue disorders	12 (0.88%)
Eye disorders	1,544 (2.75%)	Skin and subcutaneous tissue disorders	11 (0.81%)
Psychiatric disorders	1,531 (2.73%)	Investigations	8 (0.59%)
Musculoskeletal and connective tissue disorders	1,477 (2.64%)	Eye disorders	6 (0.44%)
Product issues	1,320 (2.36%)	Infections and infestations	5 (0.37%)
Infections and infestations	1,304 (2.33%)	Cardiac disorders	2 (0.15%)
Respiratory, thoracic and mediastinal disorders	1,023 (1.83%)	Renal and urinary disorders	2 (0.15%)
Renal and urinary disorders	881 (1.57%)	Blood and lymphatic system disorders	1 (0.07%)
Cardiac disorders	661 (1.18%)	Ear and labyrinth disorders	1 (0.07%)
Hepatobiliary disorders	628 (1.12%)	Hepatobiliary disorders	1 (0.07%)
Surgical and medical procedures	620 (1.11%)	Immune system disorders	1 (0.07%)
Neoplasms benign, malignant and unspecified (incl cysts and polyps)	563 (1.00%)	Vascular disorders	1 (0.07%)
Vascular disorders	477 (0.85%)		
Reproductive system and breast disorders	263 (0.47%)		
Ear and labyrinth disorders	213 (0.38%)		
Immune system disorders	213 (0.38%)		
Social circumstances	166 (0.3%)		
Endocrine disorders	162 (0.29%)		
Blood and lymphatic system disorders	109 (0.19%)		
Pregnancy, puerperium and perinatal conditions	62 (0.11%)		
Congenital, familial and genetic disorders	17 (0.03%)		

^a^ FAERS: US, Food and Drug Administration (FDA) adverse event reporting system.

^b^ SOC: system organ classes of the Medical Dictionary for Regulatory Activities.

In PTs reported, the most frequently reported were nausea, vomiting, diarrhoea, etc., consistent with the most commonly associated SOC with adverse effects being the gastrointestinal tract (Table 3). Notably, in social media, insomnia (0.74%), anxiety (0.66%), and depression (0.52%) were reported more frequently. Conversely, pancreatitis (1.03%) and blurred vision (0.67%) were more commonly reported in the FAERS database.

**TABLE 3:** Top 30 of preferred terms reported in FAERS^a^ and social media.

FAERS		Social media	
PT^b^	Freq	PT	Freq
Nausea	3,716 (6.63%)	Nausea	250 (18.4%)
Vomiting	2,119 (3.78%)	Diarrhea	129 (9.49%)
Diarrhoea	1822 (3.25%)	Hiccups	107 (7.87%)
Decreased appetite	1,456 (2.60%)	Fatigue	104 (7.75%)
Weight decreased	1,233 (2.2%)	Vomiting	101 (7.43%)
Blood glucose increased	1,078 (1.92%)	Constipation	88 (6.84%)
Constipation	1,053 (1.88%)	Abdominal pain upper	59 (4.34%)
Headache	944 (1.68%)	Flatulence	55 (4.05%)
Abdominal pain upper	827 (1.48%)	Decreased appetite	51 (3.75%)
Dizziness	751 (1.34%)	Abdominal distension	47 (3.46%)
Fatigue	737 (1.31%)	Headache	47 (3.46%)
Abdominal distension	576 (1.03%)	Gastrooesophageal reflux disease	29 (2.13%)
Malaise	575 (1.03%)	Dizziness	28 (2.06%)
Pancreatitis	575 (1.03%)	Heartburn	24 (1.77%)
Abdominal pain	567 (1.01%)	Dyspepsia	20 (1.47%)
Asthenia	532 (1.01%)	Gastrointestinal pain	18 (1.32%)
Abdominal discomfort	506 (0.95%)	Abdominal pain	17 (1.25%)
Eructation	496 (0.9%)	Abdominal discomfort	14 (1.03%)
Wrong technique in product usage process	489 (0.88%)	Decreased appetite	14 (1.03%)
Dehydration	485 (0.87%)	Insomnia	10 (0.74%)
Inappropriate schedule of product administration	485 (0.87%)	Anxiety	9 (0.66%)
Injection site extravasation	454 (0.81%)	Depression	7 (0.52%)
Blood glucose decreased	441 (0.79%)	Influenza like illness	7 (0.52%)
Product use in unapproved indication	423 (0.75%)	Lethargy	7 (0.52%)
Flatulence	419 (0.75%)	Hypoglycaemia	5 (0.37%)
Dyspepsia	414 (0.74%)	Myalgia	5 (0.37%)
Device malfunction	411 (0.73%)	Somnolence	5 (0.37%)
Vision blurred	374 (0.67%)	Alopecia	4 (0.29%)
Rash	336 (0.60%)	Back pain	4 (0.29%)

^a^ FAERS: US, Food and Drug Administration (FDA) adverse event reporting system.

^b^ PT: preferred term of the Medical Dictionary for Regulatory Activities.

### 3.3 Comparison of AEs reported in oral and subcutaneous administration

With semaglutide available in two different routes of administration, we sought to investigate potential differences in adverse reactions associated with each route. Our analysis revealed 16,711 cases of subcutaneous semaglutide and 2,508 cases of oral semaglutide. Serious outcomes, such as death and life-threatening events, accounted for 3.07% of total cases with oral semaglutide and 2.25% with subcutaneous semaglutide. At the SOC level, gastrointestinal disorders comprised 30.19% of total ADEs with oral semaglutide, slightly higher than with subcutaneous semaglutide (27.76%). But gastrointestinal toxicities were not more frequently reported in patients treated with oral semaglutide versus those treated with subcutaneous semaglutide (ROR_025_: 0.91). Further analysis indicated that the median onset days of PTs were 4 days with oral semaglutide (Q1:1, Q3:32) and the same with subcutaneous administration (Q1:1, Q3:35). Compared to oral semaglutide, subcutaneous semaglutide showed more reports of injury, poisoning, and procedural complications (11.52% vs 5.51%). The remaining SOC reports remained largely consistent between the two administration routes. Detailed information is provided in Table 4.

**TABLE 4:** SOC^a^ reported in oral and subcutaneous seaglutide.

Oral semaglutide		Subcutaneous semaglutide	
SOC	N (%)	SOC	n (%)
Gastrointestinal disorders	1792 (30.19%)	Gastrointestinal disorders	13,857 (27.76%)
General disorders and administration site conditions	580 (9.77%)	General disorders and administration site conditions	6,192 (12.41%)
Investigations	579 (9.75%)	Injury, poisoning and procedural complications	5,751 (11.52%)
Nervous system disorders	491 (8.27%)	Investigations	4,196 (8.41%)
Metabolism and nutrition disorders	359 (6.05%)	Nervous system disorders	3,545 (7.1%)
Injury, poisoning and procedural complications	327 (5.51%)	Metabolism and nutrition disorders	3,149 (6.31%)
Skin and subcutaneous tissue disorders	271 (4.57%)	Skin and subcutaneous tissue disorders	1,604 (3.21%)
Eye disorders	205 (3.45%)	Psychiatric disorders	1,392 (2.79%)
Musculoskeletal and connective tissue disorders	190 (3.2%)	Eye disorders	1,333 (2.67%)
Respiratory, thoracic and mediastinal disorders	151 (2.54%)	Musculoskeletal and connective tissue disorders	1,286 (2.58%)
Psychiatric disorders	132 (2.22%)	Product issues	1,230 (2.46%)
Renal and urinary disorders	124 (2.09%)	Infections and infestations	1,170 (2.34%)
Infections and infestations	123 (2.07%)	Respiratory, thoracic and mediastinal disorders	858 (1.72%)
Hepatobiliary disorders	94 (1.58%)	Renal and urinary disorders	753 (1.51%)
Cardiac disorders	87 (1.47%)	Cardiac disorders	565 (1.13%)
Product issues	86 (1.45%)	Surgical and medical procedures	536 (1.07%)
Surgical and medical procedures	80 (1.35%)	Hepatobiliary disorders	528 (1.06%)
Neoplasms benign, malignant and unspecified (incl cysts and polyps)	70 (1.18%)	Neoplasms benign, malignant and unspecified (incl cysts and polyps)	493 (0.99%)
Vascular disorders	54 (0.91%)	Vascular disorders	420 (0.84%)
Immune system disorders	35 (0.59%)	Reproductive system and breast disorders	234 (0.47%)
Reproductive system and breast disorders	29 (0.49%)	Ear and labyrinth disorders	195 (0.39%)
Endocrine disorders	22 (0.37%)	Immune system disorders	175 (0.35%)
Ear and labyrinth disorders	17 (0.29%)	Social circumstances	152 (0.3%)
Blood and lymphatic system disorders	14 (0.24%)	Endocrine disorders	140 (0.28%)
Social circumstances	12 (0.2%)	Blood and lymphatic system disorders	92 (0.18%)
Pregnancy, puerperium and perinatal conditions	7 (0.12%)	Pregnancy, puerperium and perinatal conditions	52 (0.1%)
Congenital, familial and genetic disorders	5 (0.08%)	Congenital, familial and genetic disorders	12 (0.02%)

^a^ SOC: system organ classes of the Medical Dictionary for Regulatory Activities.

**FIGURE 1:**
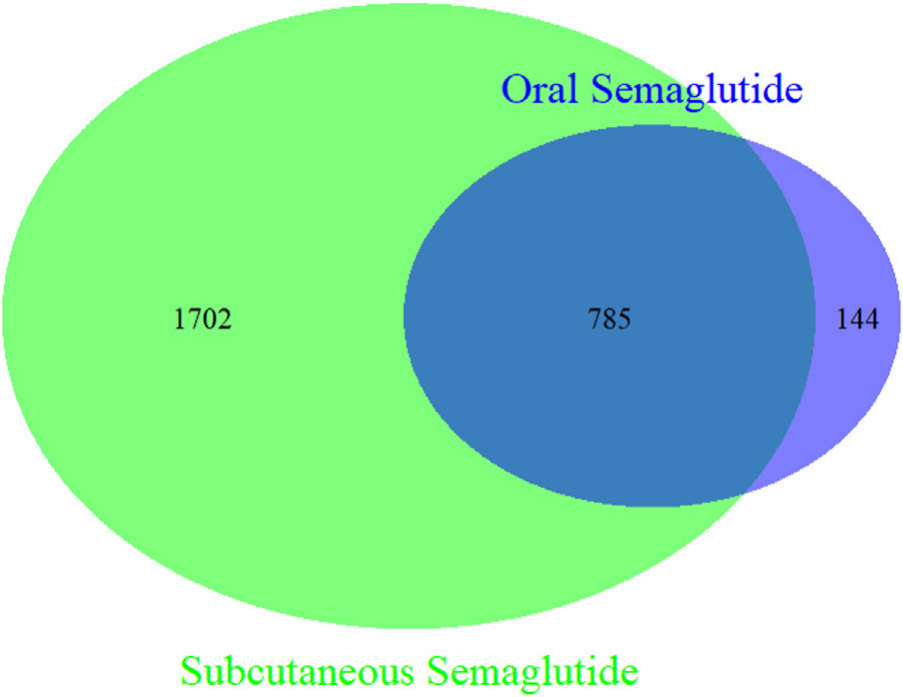
Venn diagram of PTs in oral semaglutide and subcutaneous semaglutide. Green circle: subcutaneous semaglutide. Blue circle: oral semaglutide.

**FIGURE 2:**
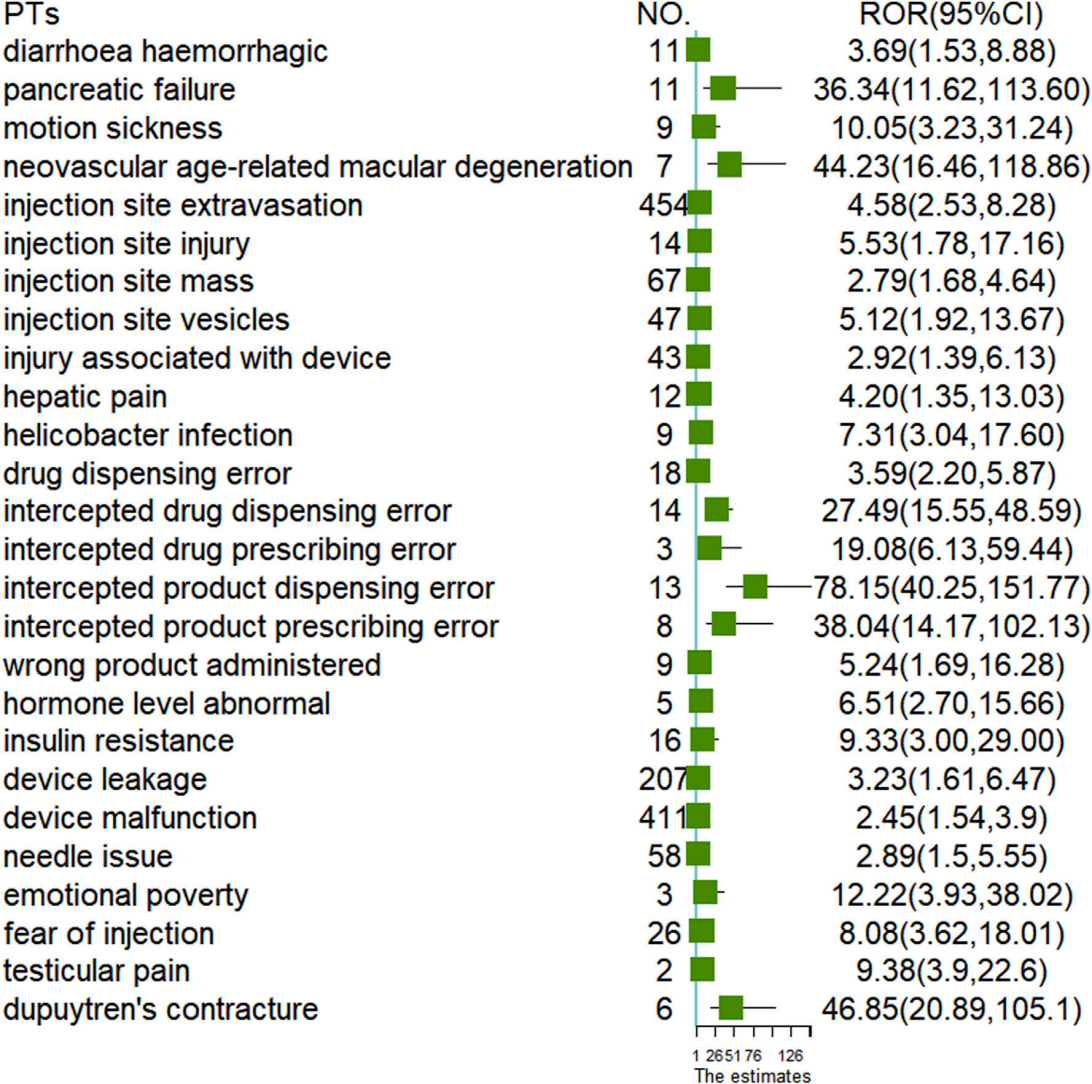
ADR signals detected in oral and subcutaneous semaglutide by disproportion analysis.

To analyze the differences at the PTs level between the two administration routes, we examined 2,508 cases of oral semaglutide and 16,711 cases of subcutaneous semaglutide. As illustrated in the Venn diagram (Figure 1), subcutaneous semaglutide reported 2,487 PTs, while oral semaglutide reported 932 PTs, with 785 PTs shared between them. Among the unique PTs reported in subcutaneous semaglutide, we identified 21 new signals through disproportionality analysis. The majority of these signals pertained to device-related issues such as leakage and malfunction, or injection-related behaviors such as injury and fear of injection. Additionally, we detected new serious AE signals including hemorrhagic diarrhea with a ROR value of 3.69 (95% CI: 1.53, 8.88), hepatic pain with an ROR value of 4.2 (95% CI: 1.35, 13.03), abnormal hormone levels with an ROR value of 6.51 (95% CI: 2.70, 15.66), and pancreatic failure with an ROR value of 36.34 (95% CI: 11.62, 113.60) in subcutaneous semaglutide. In contrast, only in oral semaglutide, we detected dupuytren’s contracture with an ROR value of 46.85 (95% CI: 20.89, 105.1). Detailed information was shown in Figure 2.

## 4 Discussion

As randomized trials examining efficacy of semaglutide were not designed to capture ADR due to small sample sizes and short follow-up. Additionally, the widespread popularity of semaglutide, particularly in the realm of weight loss, has led to a rapid increase in prescription volume. Consequently, some individuals, including those who do not medically require weight loss medication, are now using it off-label. This trend has sparked growing concerns regarding the drug’s safety profile. To gather as much data as possible for drug safety evaluation, we conducted a retrospective pharmacovigilance study by analyzing reports from both the FAERS database and health forums.

In our study, the FAERS database contained a greater variety of SOCs compared to the health forum. This discrepancy may be attributed to the significantly larger number of reports in the FAERS database, which was nearly 50 times that of the health forum. This larger data allowed for the inclusion of a wider range of ADRs. Conversely, reports in the health forum were more concentrated, with gastrointestinal disorders, general disorders and administration site conditions, respiratory, thoracic and mediastinal disorders, nervous system disorders, and metabolism and nutrition disorders accounting for 94.03% of the total reports. However, gastrointestinal disorders emerged as the most frequently reported SOC in both datasets, consistent with findings from the STEP 8 Randomized Clinical Trial, where 84.1% of participants reported gastrointestinal adverse events with semaglutide.

Besides gastrointestinal-related symptoms, mood-related symptoms such as insomnia, anxiety, and depression were more frequently reported in social media. Cases of suicidal thoughts have also been reported in the FAERS database. Approved weight loss drugs are often withdrawn from the market due to various adverse reactions, including significant concerns related to neurological safety. For example, Rimonabant was withdrawn due to reports of suicidal thoughts and actions. However, our study and others do not support a higher risk of suicidal ideation with semaglutide (Wang et al., 2024; McIntyre et al., 2024). More severe PTs (e.g., pancreatitis, blurred vision) were detected more frequently in the FAERS database. This phenomenon could be attributed to the fact that reports from social media are all self-reported, and serious adverse events may not be adequately represented in such datasets. These differences in reported adverse reactions emphasize the need for comprehensive monitoring and analysis of adverse drug reactions from diverse data sources to better understand the potential risks associated with semaglutide.

The injectable version of semaglutide was approved for use by people with diabetes in the United States in 2017, followed by the approval of the oral version in 2019. Subcutaneous semaglutide reported more PTs compared to oral formulations, likely attributed to the earlier introduction of subcutaneous preparations. Semaglutide is the only GLP-1 drug available in both oral and subcutaneous dosage forms, providing consumers with the flexibility to choose according to their own needs. The inevitable pain and discomfort caused in subcutaneous semaglutide often leads to noncompliance, resulting in patient dissatisfaction (Feng et al., 2023). Adverse reactions related to needle injuries, such as device leakage or needle issues, were associated with subcutaneous semaglutide. Patients who were afraid of injections or had experienced injection injuries could choose oral preparations instead. The number of gastrointestinal ADRs was high in both forms, but the oral dosage form did not show stronger signal compared to subcutaneous form. Further analysis was conducted to elucidate the characteristics of gastrointestinal adverse reactions associated with the two dosage forms. The results revealed that the median onset time was consistent between the two groups, indicating similar performance in terms of gastrointestinal adverse reactions. Thus, gastrointestinal adverse reactions should not be considered as a determining factor when selecting different formulations of semaglutide.

However, despite initially showing the highest persistence rate among all antiobesity medications (AOM), the persistence rate of semaglutide dropped by more than half to 40% after 1 year of treatment. Additionally, studies have reported that 1 year following the discontinuation of once-weekly subcutaneous semaglutide, individuals experienced an average weight regain of 11.6 percentage points, with many participants reverting to prediabetic states (Wilding et al., 2022). In light of ours and others findings, it becomes evident that apart from economic considerations, the intolerable gastrointestinal adverse reactions associated with GLP-1 cannot be overlooked (Sodhi et al., 2023). Dietary education targeting the reduction of gastrointestinal adverse events is paramount, may encompass instructing individuals to consume small, frequent meals as a means to alleviate nausea (Cornell, 2020). Flexibility during the dose-escalation phase is crucial, while acknowledging the potential necessity for appropriate symptomatic treatment in cases of persistent gastrointestinal adverse events.

Although our results were derived from a spontaneously reported system, the report sources revealed that 76.83% of ADR reports were submitted by health professionals, 22.24% by consumers, and 0.98% by other individuals from 2018 Q1 to 2023 Q2, indicating that the FEARS database is professional and comprehensive. The data from health forums was solely consumer-reported but underwent evaluation by two independent professionals who excluded non-compliant data. Nevertheless, there remains a potential for data bias. Additionally, it's important to note that the ROR represents the possibility of ADRs associated with semaglutide but does not necessarily reflect the true incidence of these reactions.

## 5 Conclusion

Our study delineates the safety profile of semaglutide using data from the FAERS database and social media. And compared new ADR signals and gastrointestinal performance of two dosage forms of semaglutide. Physicians should be aware of serious adverse effects to monitor patients accordingly.

## Data Availability

The original contributions presented in the study are included in the article/Supplementary Material, further inquiries can be directed to the corresponding author.

## Supplementary material

SUPPLEMENTARY TABLE S1 Examples of patients reviews of smeglutide in health forum.

SUPPLEMENTARY TABLE S2 Four grid table of disproportion analysis.
